# Study of Ni Metallization in Macroporous Si Using Wet Chemistry for Radio Frequency Cross-Talk Isolation in Mixed Signal Integrated Circuits

**DOI:** 10.3390/ma4060952

**Published:** 2011-05-25

**Authors:** Xi Zhang, Chengkun Xu, Kyuchul Chong, King-Ning Tu, Ya-Hong Xie

**Affiliations:** 1 Department of Materials Science and Engineering, University of California at Los Angeles, Los Angeles, CA 90095, USA; E-Mails: twnchong@ucla.edu (K.C.); kntu@ucla.edu (K.T.); yhx@ucla.edu (Y.X.); 2 Department of Chemical Engineering, University of Pittsburg, Pittsburgh, PA 15260, USA; E-Mail: chx9@pitt.edu

**Keywords:** RF cross-talk isolation, macroporous Si, Ni metallization

## Abstract

A highly conductive moat or Faraday cage of through-the-wafer thickness in Si substrate was proposed to be effective in shielding electromagnetic interference thereby reducing radio frequency (RF) cross-talk in high performance mixed signal integrated circuits. Such a structure was realized by metallization of selected ultra-high-aspect-ratio macroporous regions that were electrochemically etched in p^−^ Si substrates. The metallization process was conducted by means of wet chemistry in an alkaline aqueous solution containing Ni^2+^ without reducing agent. It is found that at elevated temperature during immersion, Ni^2+^ was rapidly reduced and deposited into macroporous Si and a conformal metallization of the macropore sidewalls was obtained in a way that the entire porous Si framework was converted to Ni. A conductive moat was as a result incorporated into p^−^ Si substrate. The experimentally measured reduction of crosstalk in this structure is 5~18 dB at frequencies up to 35 GHz.

## 1. Introduction

One of the major challenges for single chip radio frequency integrated circuits (RFIC’s) built on Si is the RF crosstalk through the Si substrate. Noise from switching transient in digital circuits can be transmitted through Si substrate and degrades the performance of analog circuit elements. An innovative solution to the problem was previously proposed and studied [1,2,3]. Through-the-wafer porous Si (PS) was inserted into selected regions of Si substrates. PS is an ideal material for cross-talk isolation in mixed signal integrated circuits because of its high resistivity (>10^6^ Ω-cm) [4] and its near perfect thermal expansion coefficient match to bulk Si. To further reduce crosstalk, a highly conductive moat was predicted by simulation to shield RF cross-talk [5]. The conductive moat also serves as “true ground” contacts, *i.e*., contact points on the chip surface with much reduced inductance to the true system ground. For instance, Faraday cage type of structure was demonstrated for effectively shielding RF interference and thus reducing cross-talk [6].

In the present study, we explore a novel approach for fabricating deep conductive regions within the industrial standard p^−^ Si substrate. This approach allows for complete metallization of vias with 250:1 aspect ratio. We employ a two-step process. The first step consists of selective formation of straight and parallel macropores with ultra-high-aspect-ratio into the Si substrate. Following that, we employ a Ni^2+^-contained plating bath for metallization of the pre-formed macropores at slightly elevated temperature. The method of forming macroporous Si into p^−^ Si has been studied by several research groups [7,8,9,10]. Macro-PS can be directly formed on polished p-type Si surface in organic solution containing dilute hydrofluoric (HF) acid. In the meantime, electroless plating of metals is widely utilized by the semiconductor manufacturing industry due to its simplicity, selectivity and ability of filling fine patterns [11,12]. In particular, N. Takano *et al*. introduced an aqueous nickel bath without reducing agent for Ni deposition on Si (100) aiming for fabricating fine metal dot arrays [13]. Farid A. Harraz *et al*. has studied the different Ni plating behavior from both acidic and alkaline fluoride media on the PS layer to make electrical contact for PS based devices [14]. Based on preceding work, we have developed this novel approach for RF mixed signal IC applications. Our processes are compatible with conventional Si very-large-scale integration (VLSI) technology since hydrofluoric etching and electrochemical deposition are widely used by the industry.

## 2. Results and Discussion

**Figure 1:**
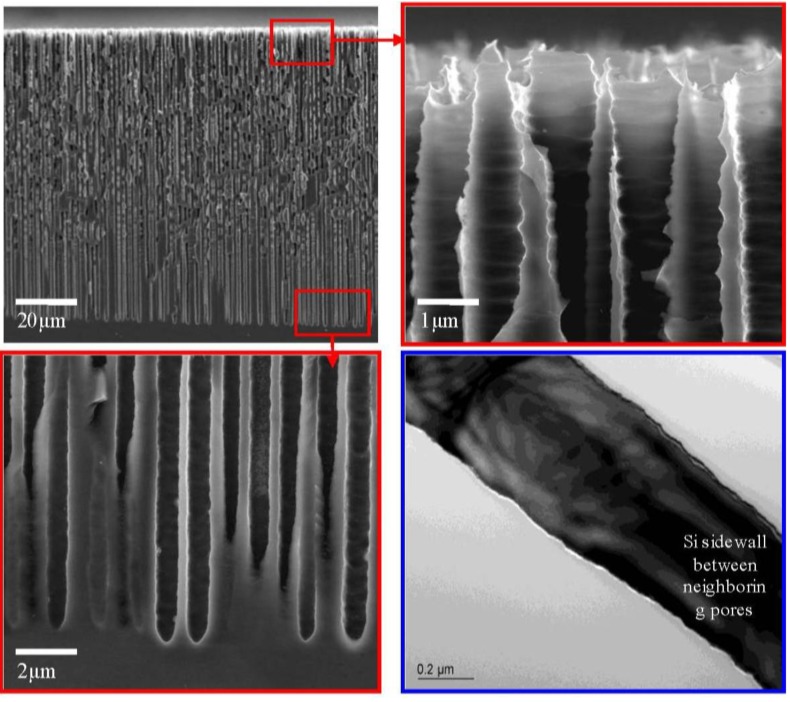
Field emission SEM (FESEM) and TEM (the lower right) micrographs of the macropores with high aspect ratio formed in p^−^ Si. Anodization was conducted in a 8% Hydrofluoric Dimethyl Sulfoxide (HF DMSO) solution with current density of 8 mA/cm^2^ applied in a two-electrode Teflon cell at room temperature.

Macropore formation in Si is understood as an anisotropic process [10]. Resultant arrayed macropores are of about 1 µm in diameter together with frame sidewall thickness being around 0.5 µm. Macropore aspect ratio is a function of time and can reach 250 with a 12-h batch process. Scanning electron microscope (SEM) and transmission electron microscope (TEM) graphs in Figure 1 show an ultra-high-aspect-ratio Si macroporous structure. These pores provide channel access for Ni^2+^ chemical solution and subsequently lead to macroporous framework metallization.

### 2.1. Metallization of Ultra-High-Aspect-Ratio Si Macropores Using Wet Chemical Plating

In our fluoride, containing alkaline aqueous electrolyte without reducing agent, the electron exchange is basically accomplished between sidewall Si atoms and solution Ni^2+^ ions although the detailed mechanism is more complicated [15,16,17]. In other words, Ni is deposited at the expense of Si through fluoridation and dissolution, also known as displacement reaction. The thermodynamic driving force comes from the difference of two electrode redox potentials that are presumably represented by the following equation [18]:

SiF_6_^2−^ (aq) + 4e^−^ → Si^0^ + 6F^−^ (aq) E^0^ = −1.20 V (SHE) (1)

Ni^2+^+2e^−^ → Ni^0^ E^0^ = −0.257 V (SHE) (2)

where E^0^ is standard electrode potential with reference to standard hydrogen electrode. It is therefore a favorable charge transfer process from Si electrode to Ni electrode. On top of that, ammonium fluoride keeps the solution in an alkaline state which results in a highly negative open circuit potential for reactive surface to kinetically drive the chemical deposition [19].

**Figure 2:**
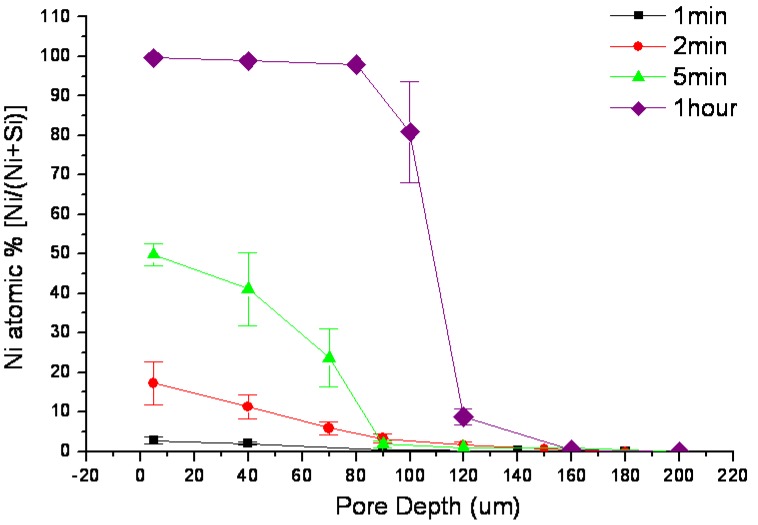
Plots of Ni atomic percentage profile measured by EDX along the pore depth for different immersion times: 1 minute, 2 minutes, 5 minutes and 1 hour.

**Figure 3:**
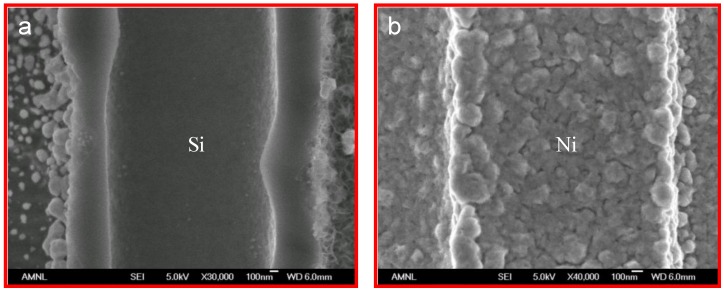
FESEM micrographs of a sample after 1-hour treatment in the Ni bath: (**a)** Pore morphology at a level where no significant deposition occurred. **(b)** Pore morphology at a level where full metallization occurred.

**Figure 4:**
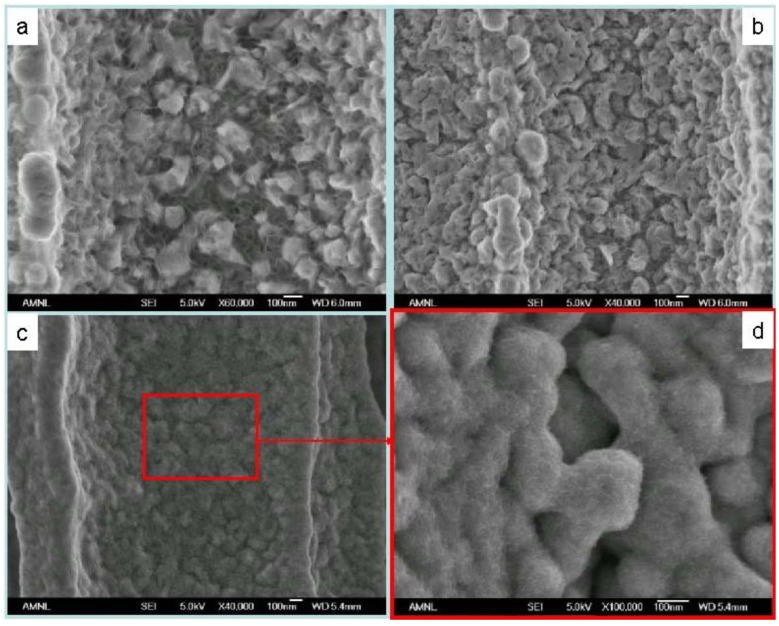
FESEM cross-sectional micrographs of the plated sidewalls at a depth of about 100 µm, taken from different samples with various degrees of treatment: **(a)** 1-h metallization stage; **(b)** 2-h metallization stage; **(c)** 8-h metallization stage; **(d)** Enlarged image of the aggregated Ni deposits.

**Figure 5:**
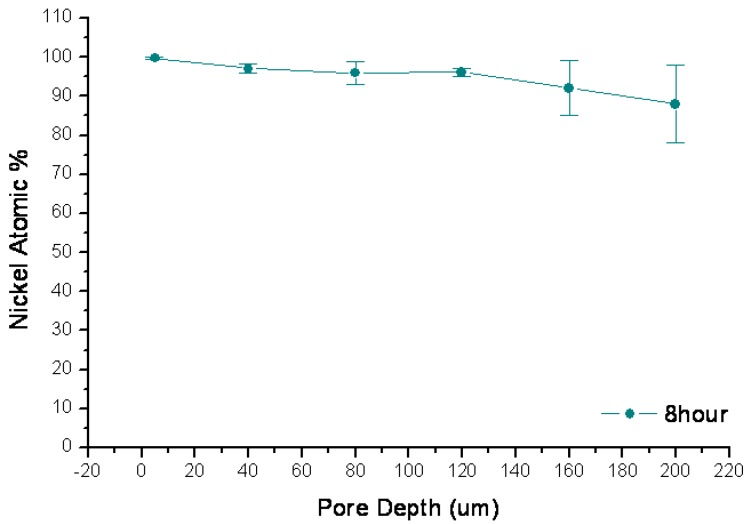
Plots of Ni atomic percentage profile measured by EDX along the pore depth for 8 hours treatment.

Porous sample cross-sections were examined by FESEM and elementally analyzed from framework top to bottom by semi-quantitative energy-dispersive X-ray spectroscopy (EDX) along longitudinal orientation in order to estimate Ni percentage of the resultant porous structure. As plotted in Figure 2, Ni^2+^ was rapidly reduced to metalize the upper portion of the macroporous skeleton by consuming Si (Si percentage decreases with extended process duration). Ni deposition was noticeably restricted on the lower portion of these pores due to limited mass transport in such high aspect ratio geometry. Cross-sectional SEM micrographs, which were taken from sidewall top and bottom locations of a 1-hour long wet-treated sample, show the contrast of surface morphologies between where little chemical deposition of Ni had occurred and where it had significantly taken place (Figure 3). Furthermore, micrographs of the plated sidewalls at a depth of about 100 µm, taken from different samples with various degrees of treatment are shown in Figure 4 revealing gradually enhanced metallic Ni coverage of sidewall surface in compliance with longer duration of immersion. Ni deposits are clustered in submicron size. Obtained from a typical 8-hour treated sample, fairly uniform Ni coverage along the longitude of 200 µm deep macroporous framework is presented in Figure 5.

**Figure 6:**
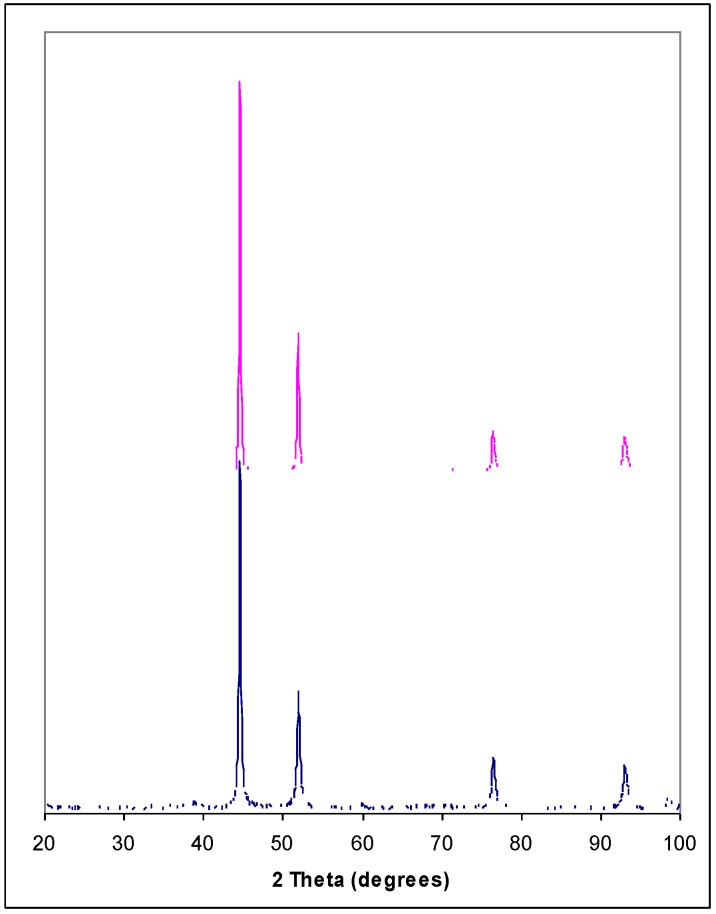
XRD spectra of pure Ni (upper) and an 8-hour wet treated sample (lower)

An interesting and important observation is that the entrance to each individual macropore remains open throughout the entire chemical process however long it was studied, *i.e*., 8 hours. This is attributed to the self-limiting nature of the deposition process. Ni^2+^ was being deposited at the expense of Si [15]. When the entire porous skeleton of Si was consumed, Ni deposition ceased. Figure 6 shows the comparison of the X-ray diffraction (XRD) θ–2θ scan results between a macroporous substrate with 8-hour immersion and a piece of pure Ni foil. It is evidenced that deposited Ni takes the form of polycrystalline in fcc configuration and major XRD peaks represent Ni (111), (200), (220), and (311). One important observation from Figure 6 is the absence of major Si (004) peaks indicative of a complete displacement leading to a macroporous Ni skeleton from the original Si.

**Figure 7:**
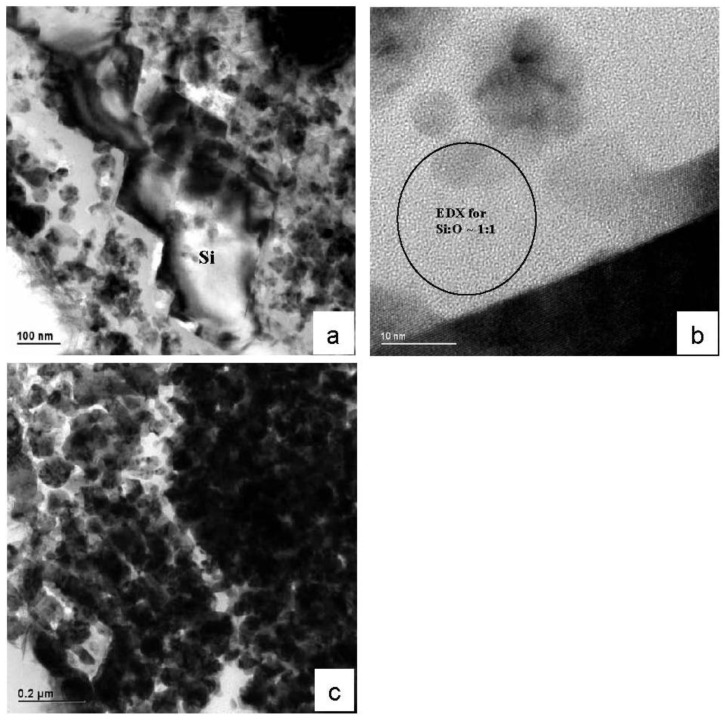
**(a)** TEM micrograph of the displacement reaction, Ni deposits, Si sidewall and the interface with (111) Si microfacets; **(b)** High-mag TEM micrograph of the reaction interface, with brightly-contrasted phase identified by EDX as Si:O ~1; **(c)** TEM micrograph of pore sidewall showing the consumption of Si and full deposition of Ni.

Deposition behaviors of various metals including Cu, Ni, Pt, Au, *etc*., on blank Si substrates in fluoride (HF or NH4F) solutions [20] have been investigated. The main work attempted to elucidate the morphological evolution, deposition kinetics and fundamentals of nucleation, growth modes and the charge transfer mechanism [21]. We have adopted a chemical bath containing concentrated Ni^2+^ ions in aqueous fluoride medium to metalize the straight sidewalls of ultra-high-aspect-ratio Si macropores. Deposition behavior can be different from that of the conventional dilute solutions. Concentration accounts for the equilibrium electrode potential of Ni^2+^ reduction and deposition rate is kinetically much enhanced by the abundance of Ni^2+^ species, because the rate determining step in high aspect ratio geometry always comes from diffusion process. It is well known that reduction requires electrons to be given by oxidizing Si atoms. It had been expected that upon one full layer deposition of Ni, displacement process would have been terminated because of no more exchange with the underlying Si. Our experimental results indicated more than that. Though a complete understanding would take further systematic electrochemical investigation, a plausible explanation can be outlined with the assistance of TEM microanalysis. Figure 7 displays TEM micrographs of the progressive sidewall reaction from a 1-hour wet chemistry treated sample. In Figure 7a, it can be seen that dark contrasted Ni were deposited into, instead of onto, the Si sidewall. This is in agreement with the displacement principle that Ni^2+^ is reduced by oxidizing and dissolving Si. Moreover, two characteristics are noticeable from the image in regard to the deposition process. Firstly, Si underwent an anisotropic etching in <111> direction, which could be related to the existence of NH_4_F in the chemistry. NH_4_F is commonly used as part of buffered oxide etching (BOE) chemicals and study of its etching and oxidation processes on Si (100) affirms increasingly generated Si (111) micro facets soon after immersion [22]. The second is that Ni deposits are not in direct contact with Si but enclosed within a matrix of bright contrast located at the interfacial area in between Ni deposits and underneath Si (Figure 7b, TEM micrograph of higher mag). Randomly spotted EDX in this bright phase, reveals composition of Si and O to approximately 1:1. This can probably be identified as certain types of Si suboxide. Formation of suboxide species at (111) micro-facets during Si etching in NH_4_F for a prolonged duration, was reported in SiH(O_3_) [23]. Regardless of the specific chemical formula, these suboxide species can, to a large extent, serve as intermediate phases or even reducing agents for Ni deposition when they experience further oxidation. Fluoride species which are strong oxidizing agents can further oxidize sub-oxide species for additional extraction of electrons to reduce Ni^2+^, and then cause dissolution of Si oxides, and eventually give rise to microporous Ni deposits on sidewalls. Some earlier detailed analysis of electrochemical kinetics and deposition chemistry in bath processes provides support to our findings [22]. Therefore transport of chemical species becomes possible through a microporous deposits layer to overreact with inner Si sidewall. Figure 7c shows the late stage of sidewall evolution in which Si was almost totally replaced by Ni deposits.

### 2.2. Cross-Talk Measurement over the Test-Structure

**Figure 8:**
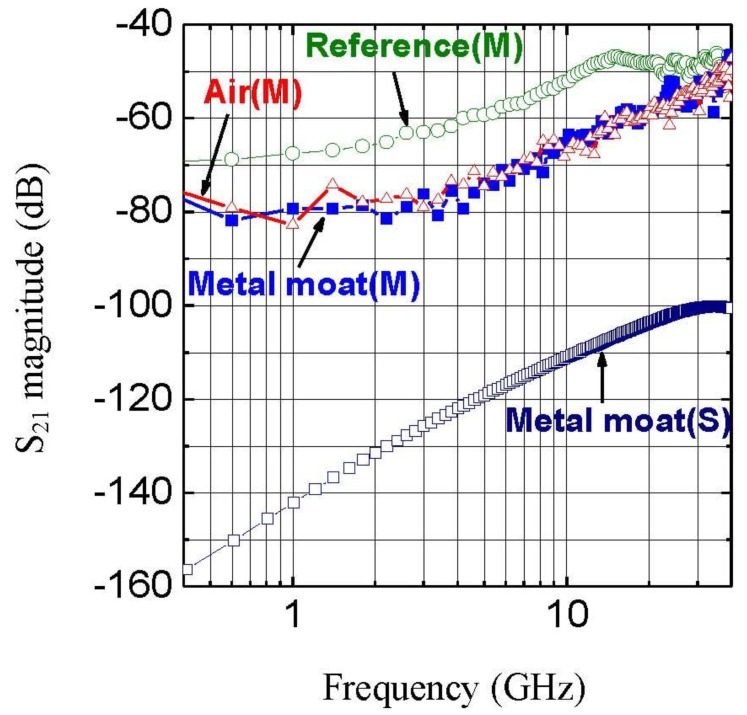
Measurement and simulation of S_21_ magnitude for the test structures with metal via isolation: Reference indicates there is no isolation structure. Air crosstalk is measured by microprobes suspended in air at Al pad separation distance of 800 µm. (M) indicates measurement result and (S) indicates simulation result.

The preceding paragraphs have demonstrated that implementation of our wet approach enables metallization of ultra-high-aspect-ratio macropores inlaid in p^−^ Si substrate serving as a highly conductive shield for suppressing cross-talk within RF mixed-signal ICs fabricated on either of its sides. Such configuration has shown promising results. Cross-talk test structure based on a p^−^/p^+^ Si substrate and detailed study was described much more thoroughly in another of our earlier publications [5] and only a brief discussion is presented here. The metalized macroporous moat through p^−^ layer has a width of 50 µm and was connected to the upper ground planes via oxide windows and the p^+^ layer beneath. The functionality of conductive moat combined with p^+^ substrate is regarded for noise bypass to the system ground. This metallic construction, due to a much reduced distance and therefore inductance to the true system ground compared with conventional ground lines that run on the front side of a chip, is ideal “true ground” contact of low inductance for a variety of digital as well as RF circuits. The main concern of our RF cross-talk issue was examined between the noise generating and noise detecting on-chip Al pads. In Figure 8, S_21_ without any isolation structures, is used as reference (Reference (M)). The experimentally measured reduction of crosstalk in this structure is 5~18 dB at frequencies up to 35 GHz (Metal moat (M)). To be highlighted, achieved RF crosstalk can be reduced to the level limited by that across the air gap between the measurement probes (Air (M)). The result indicates that the built porous metal moat can reduce the crosstalk down to the established noise floor of air in our test configuration. Its true effectiveness is however underestimated as an isolation structure. Our simulation result shows that the predicted RF cross-talk reduction using a typical metal trench can be as effective as −100 dB at 40 GHz (Metal moat (S)) [5].

For Si integrated circuit technology, it is the conventional belief that porous structures are undesirable from the point of view of long term reliability, although emerging technology such as low-k dielectrics has shown otherwise. On the other hand, the ability to transform a porous semiconductor structure into a porous metal structure as shown in this report has great potential in applications including sensors and other bio-medical devices.

## 3. Experimental Section

Experimental procedures were performed directly over the p^−^ Si substrates to seek the possibility and effectiveness in fabrication of a highly conductive region within the substrate.

### 3.1. Preparation of Macroporous Si

The p^−^ Si (100) substrate with resistivity ~10 Ω-cm was precut into 2 cm × 2 cm pieces. A two-electrode Teflon cell was used with the center 1 cm × 1 cm area of the samples exposed to the electrolyte containing 8% HF, 8% H_2_O, and 84% DMSO (Dimethyl Sulfoxide). Anodization of Si was carried out at a constant current density 8 mA/cm^2^ and at room temperature. After the electrochemical process, ethanol and pentane were used for the post-etching treatment [24].

### 3.2. Metallization of Ultra-high-aspect-ratio Si Macropores Using Wet Chemical Plating

p^−^ Si substrate with freshly etched arrays of macropores was immersed in an aqueous solution at 60 °C for wet metallization. The chemical bath (Table 1) contains a high concentration of NiSO_4_ (1.0 M) in an alkaline state [25]. The pH value was maintained at 8.0 using a buffered solution containing NH_4_OH and (NH_4_)_2_SO_4_. Minute amount of wetting agent was added to provide adequate wetting of sample surface and avoid blistering due to gas bubbles. Instead of using a reducing agent, NH_4_F was added to promote the chemical deposition. This is a common method when working with semiconductor surfaces [20]. In addition, complexing agent was excluded since NH_4_OH already bears good complexing ability with Ni^2+^ ions [14]. Both pH value and working temperature were carefully monitored and maintained. Samples were immersed for 1 minute, 2 minutes, and 5 minutes, 1 hour and 8 hours. Cross-sectional samples were prepared and examined by X-ray diffractometer (XRD, Simons), field emission scanning electron microscope (FESEM, JOEL) and transmission electron microscope (TEM, Philips), equipped with energy dispersive X-ray spectrometers (EDX, Oxford). Samples for TEM observation were manually sectioned and further thinned by ion milling to approximately 100 nm thick.

**Table 1:** Ni plating bath compositions and operating conditions.

*Major Chemicals*	*Moles per Liter (M)*
NiSO_4_ 6H_2_O	1
(NH_4_)_2_SO_4_	0.5
Reducing/Complexing agents	0
Wetting agent	10 mg in 50 mL bath
*Conditions*	
pH = 8.0 adjusted by Ammonia	Temperature = 60 °C

### 3.3. Cross-Talk Measurement over the Test-Structure

As a result, we made use of this approach to incorporate the resultant moat into the p^−^ part of the Si substrate in our test circuit structure for cross-talk isolation [5]. Two port S-parameters were measured using a HP8722ES network analyzer up to 40 GHz. Parameter S_21_ without any isolation structure is used as reference. Cross-talk through the air is measured as a second reference by microprobes suspended in air.

## 4. Conclusions

In conclusion, we have used a method of wet Ni^2+^ chemistry to metalize the inlaid region of p^−^ Si with pre-etched arrays of ultra-high-aspect-ratio macropores for building a highly conductive moat structure in Si substrate for cross-talk reduction in a single RF mixed-signal IC chip. Ni deposition takes place based on the mechanism of electron exchange between Si and Ni^2+^. Such a reaction is initiated as transport-limited and ended in a mode with Si being replaced by Ni deposits on sidewalls of macroporous skeleton. In other words, the approach converts macroporous Si to a structure composed of Ni. It helps to realize a highly conductive region in Si substrate and offer potential applications in mixed-signal integrated circuits. The experimentally measured reduction of crosstalk in the engineered substrate structure is 5~18 dB at frequencies up to 35 GHz.
